# Model Catanionic Vesicles from Biomimetic Serine-Based Surfactants: Effect of the Combination of Chain Lengths on Vesicle Properties and Vesicle-to-Micelle Transition

**DOI:** 10.3390/membranes13020178

**Published:** 2023-02-01

**Authors:** Isabel S. Oliveira, Sandra G. Silva, Maria Luísa do Vale, Eduardo F. Marques

**Affiliations:** 1 CIQUP, IMS (Institute of Molecular Sciences), Departamento de Química e Bioquímica, Faculdade de Ciências, Universidade do Porto, Rua do Campo Alegre s/n, 4169-007 Porto, Portugal; 2 LAQV-REQUIMTE, Departamento de Química e Bioquímica, Faculdade de Ciências, Universidade do Porto, Rua do Campo Alegre s/n, 4169-007 Porto, Portugal

**Keywords:** amino acid-based surfactants, catanionic vesicles, self-assembly, asymmetric chain lengths, drug/gene delivery

## Abstract

Mixtures of cationic and anionic surfactants often originate bilayer structures, such as vesicles and lamellar liquid crystals, that can be explored as model membranes for fundamental studies or as drug and gene nanocarriers. Here, we investigated the aggregation properties of two catanionic mixtures containing biomimetic surfactants derived from serine. The mixtures are designated as 12Ser/8-8Ser and 14Ser/10-10Ser, where mSer is a cationic, single-chained surfactant and n-nSer is an anionic, double-chained one (m and n being the C atoms in the alkyl chains). Our goal was to investigate the effects of total chain length and chain length asymmetry of the catanionic pair on the formation of catanionic vesicles, the vesicle properties and the vesicle/micelle transitions. Ocular observations, surface tension measurements, video-enhanced light microscopy, cryogenic scanning electron microscopy, dynamic and electrophoretic light scattering were used to monitor the self-assembly process and the aggregate properties. Catanionic vesicles were indeed found in both systems for molar fractions of cationic surfactant ≥0.40, always possessing positive zeta potentials (*ζ* = +35–50 mV), even for equimolar sample compositions. Furthermore, the 14Ser/10-10Ser vesicles were only found as single aggregates (i.e., without coexisting micelles) in a very narrow compositional range and as a bimodal population (average diameters of 80 and 300 nm). In contrast, the 12Ser/8-8Ser vesicles were found for a wider sample compositional range and as unimodal or bimodal populations, depending on the mixing ratio. The aggregate size, pH and zeta potential of the mixtures were further investigated. The unimodal 12Ser/8-8Ser vesicles (<*D*_H_> ≈ 250 nm, pH ≈ 7–8, *ζ* ≈ +32 mV and a cationic/anionic molar ratio of ≈2:1) are particularly promising for application as drug/gene nanocarriers. Both chain length asymmetry and total length play a key role in the aggregation features of the two systems. Molecular insights are provided by the main findings.

## 1. Introduction

Model membrane systems, like liposomes, lamellar liquid crystals, supported lipid bilayers and Langmuir monolayers, have been extremely relevant for fundamental studies related to the physiological and biophysical properties of biological membranes [1,2,3]. They have also become very useful in terms of biomedical and pharmaceutical applications, chief among them the development of efficient nanocarriers for bioactive molecules [4]. While many studies have involved model systems composed of natural amphiphiles, like phospholipids and lipid/bile salt mixtures [5,6,7], in recent decades, colloid science has unveiled many synthetic molecules that also have bilayer-forming properties, such as double-tailed surfactants, nonionic surfactants at high temperature, and block copolymers (e.g., poloxamers) [8,9,10,11]. These synthetic amphiphiles frequently have the advantage of being chemically stable (no enzymatic degradation nor hydrolysis) and cost-effective compared to natural polar lipids, which is an advantage when fundamental studies are sought. However, for applications in nanomedicine and cosmetics a critical drawback is that the synthetic molecules often pose problems of poor biocompatibility (e.g., ocular or skin irritation and high cytotoxicity levels).

Among the colloidal systems prone to the formation of model bilayers are the so-called catanionic mixtures [12,13,14]. They consist in mixtures of a cationic and an anionic surfactant, combined in different mixing ratios and with a fixed overall concentration. Catanionic mixtures have attracted interest in the last two decades due to their particular properties. These systems typically display pronounced synergism in interfacial properties compared to the individual components, namely enhanced surface adsorption and very low values of critical micelle concentration [14,15]. In terms of aggregation, under appropriate compositions, they can also give rise to stable bilayer aggregates, like vesicles, even if the individual surfactants do not form them [12,16,17]. Moreover, strong electrostatic and chain–chain interactions between each moiety typically lead to the formation of a new type of compound (the salt-free catanionic surfactant), often with properties that are notably different from those observed for the neat surfactants [18,19,20,21,22]. For instance, catanionic surfactants commonly self-assemble to lamellar phases in a fashion akin to zwitterionic lipid bilayers, having helped to understand the repulsive hydration force between zwitterionic lipid bilayers [9,23].

The wide diversity of commercially available surfactants has provided a solid basis to generate many catanionic systems and catanionic vesicles, leading to the performance of extensive fundamental studies [24,25,26,27]. Catanionic vesicles have been shown to possess particularly appealing features, namely facile preparation, high colloidal stability (in some cases, true thermodynamic stability) and versatile structural properties (e.g., controllable size and net charge) [13,28]. Thus, they have been successfully employed as drug [29,30,31,32,33,34,35] and nucleic acid [36,37,38] delivery systems (in vitro), as well as nano-reactors for particle synthesis [39,40]. For biomedical applications, it is important that the catanionic vesicles exhibit a priori a good biocompatibility profile and thus numerous studies have evaluated their toxicity profiles [41,42,43,44,45,46,47]. Many types of biofriendly surfactants that mimic lipid behavior, and that have the potential to form biocompatible catanionic vesicles, have been developed in the last two decades [48,49,50]. The use of natural motifs, such as sugar [49,51], fatty acid [52] or amino acid moieties, as headgroups [53,54,55,56,57,58] has been shown to be a viable path for these biomimetic surfactants.

**Figure 1:**
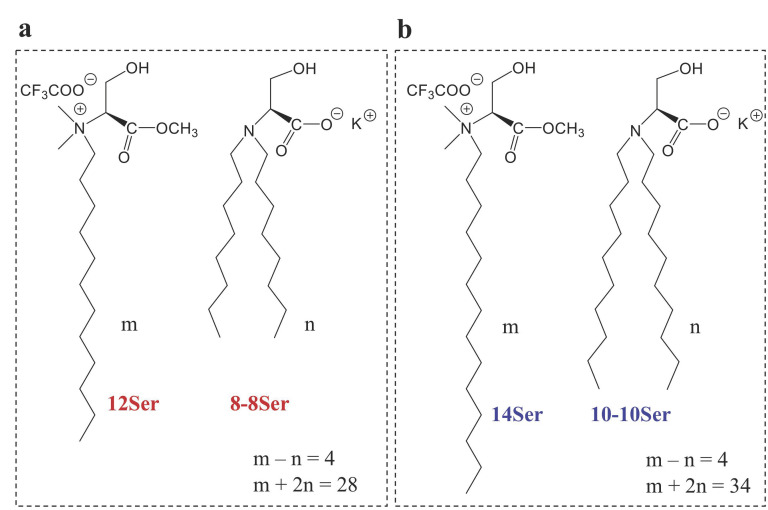
Molecular structure of the serine-based surfactants that form the catanionic mixtures: (**a**) 12Ser/8-8Ser system; (**b**) 14Ser/10-10Ser system. The letters n and m represent the number of C atoms in the hydrocarbon chains; m − n is thus the chain length asymmetry and m + 2n is the total chain length.

In particular, we previously reported that serine residues, conveniently made as cationic or anionic polar headgroups, offer a good toxicological profile for surfactants [55,56,59,60]. We prepared mixtures that can be generally designated as mSer/n-nSer, where mSer is a cationic, single-chained surfactant and n-nSer is an anionic, double-chained one (m and n being the C atoms in the chain). Specifically, the 16Ser/8-8Ser mixture, characterized by a high asymmetry of alkyl chain lengths of its constituents, is particularly interesting for generating catanionic vesicles with varying size, charge and pH, and excellent capacity to encapsulate and deliver doxorubicin to tumor cells [28,30]. Conversely, the 12Ser/12-12Ser mixture leads to solid/liquid phase separation and no ability to form vesicles at room temperature [28]. Here, we expand these studies and explore other serine-based systems, with asymmetry not so pronounced as the 16Ser/8-8Ser system (m − n = 8), namely the 12Ser/8-8Ser and the 14Ser/10-10Ser systems shown in Figure 1 (both with m − n = 4). Our main goal is to explore the effects of chain length asymmetry (fixed at 4) and total chain length (28 vs. 34 carbon atoms) on the formation of catanionic vesicles. More particularly, we intend to evaluate the effect of the combinations of chain lengths on both the vesicle/micelle transition and the characteristics of the catanionic vesicles, especially their region of existence, size, polydispersity, zeta potential and pH.

## 2. Materials and Methods

### 2.1. Synthesis

**Scheme 1:**
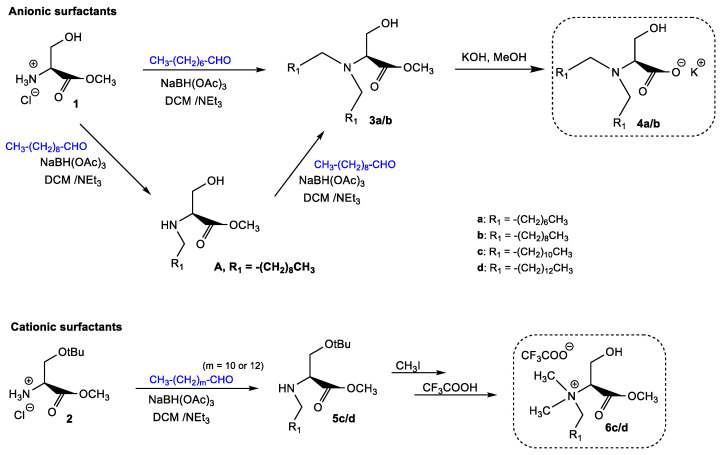
Synthetic routes for the serine-based surfactants.

The serine-based surfactants were synthesized according to procedures previously developed by our group [28,59]. The synthetic pathway to obtain these compounds is outlined in Scheme 1, and will be briefly described. Two anionic surfactants with different alkyl chains were synthesized: K8-8Ser (3a) and K10-10Ser (3b). The introduction of the alkyl chains into serine derivative 1 was achieved by reductive amination; however, it was observed that, depending on the alkyl chain length of the aldehyde, the introduction of the two alkyl chains into 1 occurred either in one step or required two consecutive reductive aminations (for alkyl chains with 10 or more carbon atoms). Accordingly, reductive amination of octanal with the serine derivative (1) yielded the dialkylated precursor methyl *N*,*N*-dioctylserinate (3a) in one step with 95% yield. On the other hand, reductive amination of decanal afforded the *N*-monoalkylated compound (A), which was reacted again with decanal to yield the *N*,*N*-didecylserine methyl ester (3b). The anionic surfactants 4a/4b were readily accessed by saponification of the corresponding *N*,*N*-dialkylserine methyl esters (3a/3b) with KOH/MeOH. 

For the synthesis of the cationic surfactants, the serine derivative 2 was used as starting material. The first step in the reaction sequence consisted in the transformation of 2 into the corresponding *N*-alkylated precursor (5c/5d) by reductive amination, using dodecanal or tetradecanal. Exhaustive methylation of these compounds, followed by removal of the *tert*-butyl protecting group yielded the target single chained surfactants 6c/6d. 

All the compounds were identified by ^1^H/^13^C NMR spectroscopy and mass spectrometry data, which is in accordance with previous reports [28,59]. Spectroscopic data for compound 4b, which was synthesized here for the first time, are as follows:

Potassium *N*,*N*-didecylserinate (4b): white powder (82%). ^1^H NMR (CD_3_OD, 400 MHz): δ 4.12 (dd, *J* = 13.0, 3.8 Hz, 1H, -CH(H)OH), 4.03 (dd, *J* = 13.2, 7.2 Hz, 1H, -CH), 3.85 (dd, *J* = 7.2, 3.6 Hz, 1H,-CH(H)OH), 3.35–3.20 (m, 4H, 2x (-N-CH_2_-)), 1.83–1.67 (m, 4H, 2 x (-N-CH_2_-CH_2_-)), 1.44–1.23 m, 28H, 2 x (-CH_2_-)_7_), 0.90 (t, *J* = 6.8 Hz, 2 x –CH_3_, 6H). ^13^C NMR (CD_3_OD, 75 MHz): δ 170.9 (C=O), 69.2 (-CH), 59.7 (-CH_2_OH), 53.7 (-N-CH_2_-), {33.1, 30.6(4), 30.5(6), 30.4(6), 30.2, 27.7, 25.8, 23.8 (-CH_2_-)_8_)} and 14.5 (-CH_3_).

### 2.2. Sample Preparation

The catanionic samples were prepared by directly weighing the two respective surfactants into vials and adding Milli-Q^®^ ultrapure water followed by thorough mixing of all the components by vigorously stirring for at least 24 h until the phase behavior could be properly established. The composition of the samples is expressed by surfactant weight percentage and molar fraction of cationic surfactant (mSer) in the mixture, as further defined in Section 3.1. In cases where solutions were formed, the surfactant concentration is expressed in molarity.

### 2.3. Video-Enhanced Light Microscopy (VELM)

To establish the phase behavior and detect colloidal aggregates (>1 µm), VELM imaging of the samples was performed with an Olympus BX51 (Tokyo, Japan) microscope using bright-field illumination with differential interference contrast (DIC). The images were acquired with an Olympus DP71 digital video camera and the micrographs were processed with the Olympus CellA software provided by the manufacturer. The microscope was also used in polarized light mode to capture any birefringent structures (e.g., Maltese crosses, indicative of large multilamellar vesicles).

### 2.4. Cryo-Scanning Electron Microscopy (Cryo-SEM)

Solutions containing colloidal aggregates (namely vesicles) were vitrified and imaged with a JEOLJSM 6301F high-resolution scanning electron microscope, equipped with a Gatan Alto 2500 preparation chamber. Each sample was placed in a copper sample-holder and vitrified by plunging it from room temperature into liquid-nitrogen slush. The vitrified sample was then transferred to the preparation chamber to be fractured, sublimated at −90 °C (for 120 s) and sputtered with an Au/Pd coating (for 40 s) to render it electrically conductive. Afterwards, it was transferred to the microscope for imaging in secondary electron (SE) mode.

### 2.5. Dynamic (DLS) and Electrophoretic (ELS) Light Scattering

Measurements of particle size (by DLS) and zeta potential, *ζ*, (by ELS) were performed with a Zetasizer Nano ZS from Malvern Instruments. A 4 mW He-Ne laser (operating at 633 nm) was used with a fixed scattering angle of 173° (for particle size) or 17° (for zeta potentials). For both DLS and zeta potential measurements, 5 repeats per sample (minimum) were performed. The autocorrelation function was analyzed using the Malvern Dispersion Technology Software (DTS), with multiple narrow mode (high resolution) data processing. Given the multimodal or polydisperse unimodal character of the observed distributions, a non-negative least squares (NNLS) algorithm was employed [61,62]. The electrophoretic mobility, *μ*, was measured using electrophoresis and laser Doppler velocimetry techniques, and ζ was calculated from μ using the Henry equation, with a dielectric constant of 78.5, a medium viscosity of 0.89 cP, and a f(*κa*) function value of 1.5 (Smoluchowsky approximation) [61,62]. 

### 2.6. Surface Tension

The surface tension of either neat surfactant or catanionic solutions was measured with a DCAT 11 tensiometer from Dataphysics^®^ using a Whilelmy Pt/Ir plate. The temperature was kept constant using a Julabo^®^ thermostated circulating bath, at 25.0 ± 0.1 °C. 

## 3. Results and Discussion

### 3.1. Phase Behavior and Aggregate Structure for the Catanionic Mixtures

**Figure 2:**
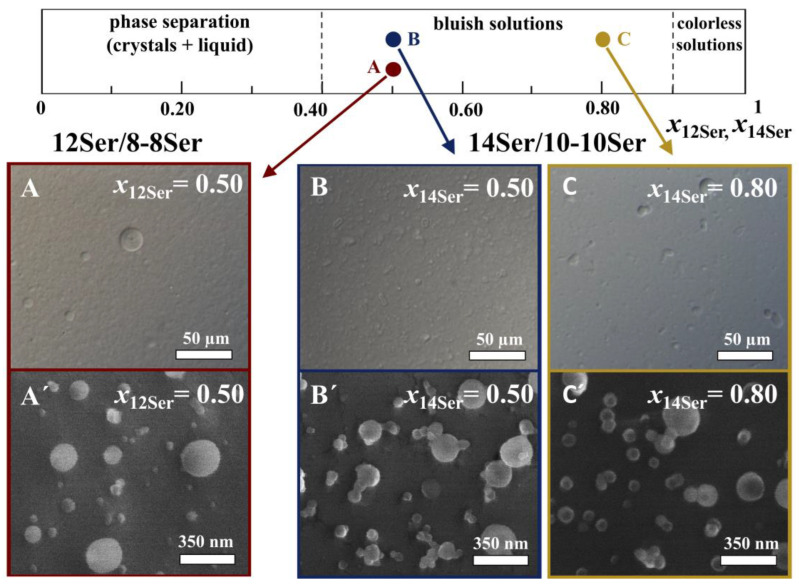
Phase maps and aggregate imaging of the 12Ser/8-8Ser and14Ser/10-10Ser catanionic mixtures, at 0.5 wt% total surfactant and 25.0 °C; *x*_12Ser_ and *x*_14Ser_ represent the molar fraction of the respective cationic surfactant in the mixture. (**A**–**C**) are VELM micrographs showing giant µm-sized vesicles; (**A′**–**C′**) are cryo-SEM micrographs.

The phase behavior and aggregation properties of the two catanionic mixtures were initially investigated and the results are shown in Figure 2. A few considerations should be made first. The individual anionic surfactants, 8-8Ser and 10-10Ser, were only soluble in water at 25 °C in alkaline conditions, due to the hydrolysis equilibrium of the surfactants and partial protonation leading to the insoluble acid form, a process that can be illustrated by the simplified equation RCOO^−^ (aq) + H_2_O (l) → RCOOH (s) + OH^−^ (aq). For this reason, the pH had to be adjusted to 12 with KOH to remove all the precipitated acid. Additionally, the Krafft temperature of the cationic surfactant 14Ser was measured as ≈26–27 °C. However, when investigating the 12Ser/8-8Ser and 14Ser/10-10Ser mixtures, it was realized that both originated extensive solution regions without any previous pH adjustment and at room temperature. Thus, the phase studies were performed at 25 °C and non-adjusted pH, employing direct ocular observations, VELM and cryo-SEM, and looking mainly for the formation of discrete colloidal aggregates (and, in particular, vesicles) in solution. The samples were prepared at a constant 0.5 wt% total surfactant concentration—i.e., 99.5 wt% water, meaning these were highly diluted samples—and with varying proportions between the surfactants. The latter are expressed as the molar fraction of cationic surfactant, defined as *x*_mSer_ = *n*_mSer_/(*n*_mSer_ + *n*_n-nSer_), where *n* is the number of moles of surfactant. Alternatively, we also use the molar ratio between surfactants, defined as *R*_+/−_ = *n*_mSer_/*n*_n-nSer._

Both systems show common features in terms of macroscopic phase behavior, as depicted in Figure 2. Firstly, three main phase regions could be identified. For *x*_mSer_ ≤ 0.40, i.e., for excess double-chained anionic surfactant, phase separation occurs in the form of a crystalline solid precipitating out of solution. This solid is most likely the 1:1 stoichiometric complex (catanionic surfactant), since when these samples were heated up to 80 °C, it remained totally insoluble; however, no further investigations on this issue were performed. Significantly, for 0.40 < *x*_mSer_ ≤ 0.90, a rather wide composition range, solutions with a bluish hue (Tyndall scattering) form, indicating the presence of large aggregates. For *x*_mSer_ > 0.90, where there is a high excess of cationic surfactant, colorless and weakly scattering solutions appear (small aggregates present). Secondly, microscopy imaging of the intermediate bluish solutions of both systems clearly show that the large aggregates are vesicles. Representative micrographs A–C (VELM) and A′–C′ (cryo-SEM) are provided in Figure 2. The vesicles in both systems are very polydisperse, as the diameters range from roughly 20–400 nm, as imaged by cryo-SEM, to ca. 10–20 µm, as captured directly by VELM. Finally, in both systems, the regions of higher interest were those around equimolarity and with excess cationic surfactant, as no phase separation was observed. This means that the single-chained cationic surfactant plays a pivotal role in stabilizing soluble colloidal aggregates, in a similar way to the previously reported 12Ser/12-12Ser and 16Ser/8-8Ser systems [28].

Having established the presence of catanionic vesicles in the two mixtures, it was important to answer several other questions: (i) what are the vesicle size distribution, vesicle charge, and the pH of these solutions?; (ii) are vesicles the only aggregates present in the bluish solution region?; (iii) knowing a priori that the neat cationic surfactants self-assemble into micelles, how does the vesicle-to-micelle transition progress as cationic surfactant is added?; (iv) what is the critical aggregation concentration of vesicle-containing solutions? In order to address these questions, DLS and surface tension measurements were carried out, and the results and insight withdrawn are presented in the next two sections.

### 3.2. Vesicle Characterization: Size, Charge, and pH

**Figure 3:**
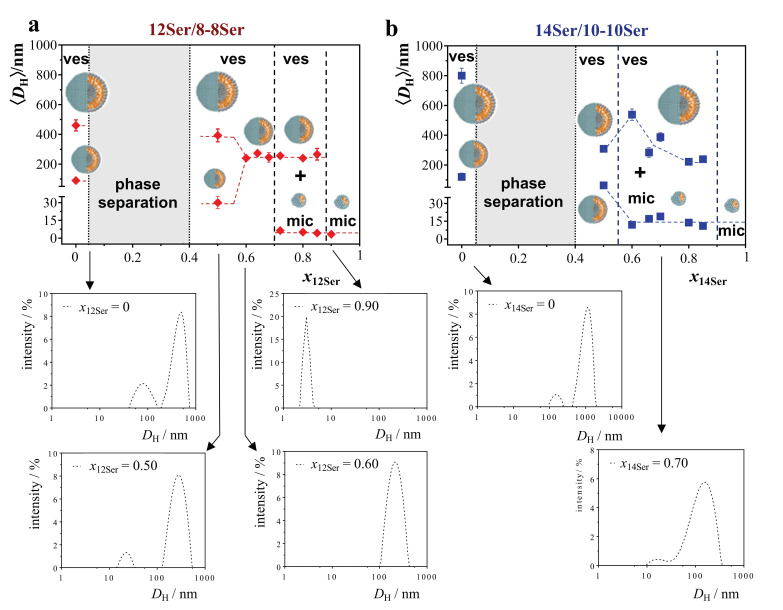
Plots of the average hydrodynamic diameter of the aggregates as measured by DLS vs. molar fraction of cationic surfactant for the two mixtures: (**a**) 12Ser/8-8Ser and (**b**) 14Ser/10-10Ser. For some compositions, the DLS (intensity-weighted) size distributions are shown: *x*_Ser12_ = 0, 0.50, 0.60 and 0.90 for 12Ser/8-8Ser; *x*_Ser14_ = 0 and 0.50 for 14Ser/10-10Ser.

The results of DLS measurements, performed in the solution regions of the two catanionic systems, are shown in Figure 3. In the two upper graphs, the average hydrodynamic diameter of the aggregates, <*D*_H_> (± standard deviation), is plotted vs. molar fraction of cationic surfactant. Below each plot, representative intensity-weighted size distributions from DLS are provided to illustrate how the upper plots were constructed and to make comparisons. 

While the aggregation behavior is similar between the two systems up to a molar fraction of cationic surfactant of ≈ 0.40, there are a few key differences in the region 0.40–1. Analyzing first the 12Ser/8-8Ser system, vesicles are the single form of assembly in the approximate range 0.40 < *x*_12Ser_ < 0.70. It is only between ≈ 0.70 and ≈ 0.88 that they are seen coexisting with micelles, detected in the DLS as aggregates of ca. 4–6 nm. Moreover, and interestingly, within the vesicle region, the population is bimodal for *x*_12Ser_ ≈ 0.40–0.60 (large vesicles with <*D*_H_> ≈ 400 nm and small ones of ≈ 30 nm) but become unimodal, with <*D*_H_> ≈ 250 nm, in the narrow region of 0.60 < *x*_12Ser_ < 0.70; these distributions remain stable for at least 6 months. Additionally, and significantly, the vesicles of the 0.70–0.88 coexistence region remain constant in size at ≈ 250 nm. This strongly suggests that they have a particular average composition responsible for their stability, corresponding to *x*_12Ser_ ≈ 0.65 or *R*_+/−_ ≈ 2:1. We will come back to this point in the discussion Section 3.4.2 below.

In the 14Ser/10-10Ser system, three important differences arise. First, vesicles as single aggregates are found only for an extremely narrow composition region (≈0.40–0.55) and as a bimodal population (≈80 and 300 nm). Secondly, though the vesicles coexist with the micelles for the entire 0.60–0.90 range, the vesicle average size is not constant, instead decreasing with increasing *x*_14Ser_. Contrary to the previous system, this supports the notion that the vesicle composition changes with the sample composition. Thirdly, the coexisting micelles have an average size ranging between 11 and 19 nm, more than twice as large as that of the 12Ser/8-8Ser mixed micelles. This result is qualitatively unsurprising, given that the chain lengths involved in the 14Ser/10-10Ser are larger, and thus should lead to larger cross-sectional lengths, irrespective of the exact micelle shape. Overall, the picture that emerges is that the 14Ser/10-10Ser mixture is more polymorphic and polydisperse than the 12Ser/8-8Ser, given that for the former the vesicle/micelle region is wider, the vesicles change with mixing ratio and single vesicles are only found as bimodal populations. The deeper significance of these results will be further discussed in Section 3.4.

**Figure 4:**
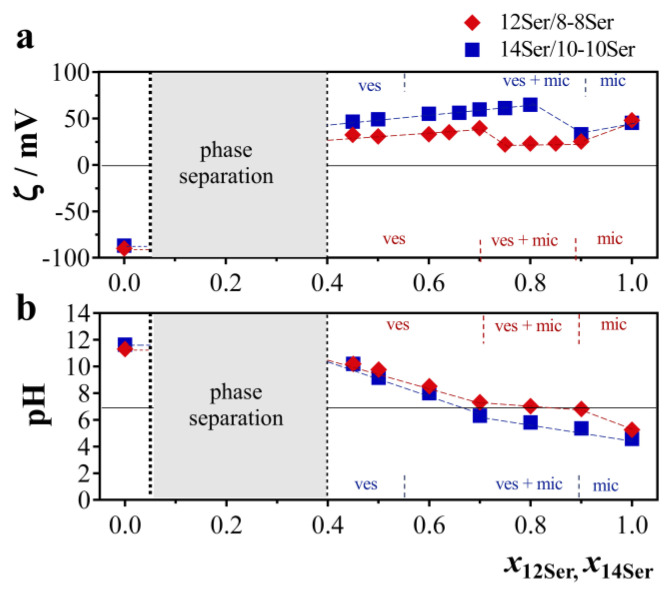
Zeta potential (**a**) and pH (**b**) of 12Ser/8-8Ser and14Ser/10-10Ser catanionic mixtures, at 0.5 wt% total surfactant and 25 °C.

Analyzing now the zeta potential and pH plots of Figure 4, further interesting features appear. The trends in the variation of *ζ* and pH are essentially similar between the two mixtures, but some subtle differences also surface. The *ζ* values in these systems depend on several varying factors, in an intricate way: system composition, size and shape of the aggregates, pH and ionic strength. Regarding the pH, it is affected by the acid/base equilibrium of the n-nSer surfactants, by the intrinsic acidic pH of the cationic surfactants (as shown by the pH at *x*_mSer_ = 1), and presumably also by the aggregation state of the system. A detailed and unequivocal analysis of the results is virtually impossible, yet some trends can be qualitatively withdrawn and rationalized.

The neat 8-8Ser and 10-10Ser vesicles (both bimodal, Figure 3a,b) are strongly negatively charged, as denoted by *ζ* ≈ −90 mV; they are also alkaline, with a pH ≈ 11–12 (a consequence, in this particular case, of the previous pH adjustment with KOH, as mentioned in Section 3.1). However, the catanionic vesicles are for all system compositions net positively charged: for the 12Ser/8-8Ser vesicles (*x*_12Ser_ = 0.40–0.70) *ζ* is roughly constant and of about +32 mV, while for the 14Ser/10-10Ser vesicles it is larger, +47 mV, increasing smoothly in the vesicle/micelle region up to +65 mV at *x*_14Ser_ = 0.80. This increase is not surprising, and essentially reflects the increase in cationic surfactant content in the systems. Yet, for the 12Ser/8-8Ser system, *ζ* drops to about +22–23 mV in the *x*_12Ser_ = 0.70–0.88 region, even though the system’s cationic content is still increasing; this decrease seems to reflect the deep morphological change in the system on going from vesicles to coexisting vesicles and micelles. A fall-out in *ζ* also takes place (even more sharply) in the 14Ser/10-10Ser, but at *x*_14Ser_ ≥ 0.90, where basically only highly cationic micelles should be present. Hence, we can conclude that aggregate size, shape, and coexistence strongly affect *ζ* in these systems.

If we turn to the pH evolution, we see that the pH decreases smoothly from alkaline (9–10) to acidic (≈5) in both systems, with neutral pH occurring for roughly *x*_14Ser_ ≈ 0.70. We recall that there was no a priori pH adjustment of the n-nSer solutions, and so these values reflect the acid/base equilibria taking place naturally.

A striking feature is that even the equimolar (*R*_+/−_ = 1, *x*_mSer_ = 0.50) vesicles in both systems are, contrary to expectation, strongly cationic, specifically with *ζ* = +31 mV for the 12Ser/8-8Ser and *ζ* = +49 mV for the 14Ser/10-10Ser vesicles, whereas one might have expected values near 0 mV. It can be seen from Figure 4b that, also somewhat surprisingly, they have an alkaline pH (9–10). These two facts in combination are quite significant. The alkaline pH most likely stems from partial protonation of the carboxylate group present in the headgroup of 8-8Ser and 10-10Ser. This means that in the vesicles, the anionic component is in reality in a RCOO^−^/RCOOH equilibrium, with a consequent contribution to an increase in *ζ*. We cannot exclude that there could be a differential composition between the outer and inner monolayer, a hypothesis proposed previously for the stabilization of catanionic vesicles [63]. An inner monolayer more enriched in the double-chained surfactant (for reasons of negative mean curvature adjustment) would obviously mean more (cationic) single-chained surfactant in the outer layer, and hence a positive zeta potential.

### 3.3. Critical Aggregation Concentrations

**Figure 5:**
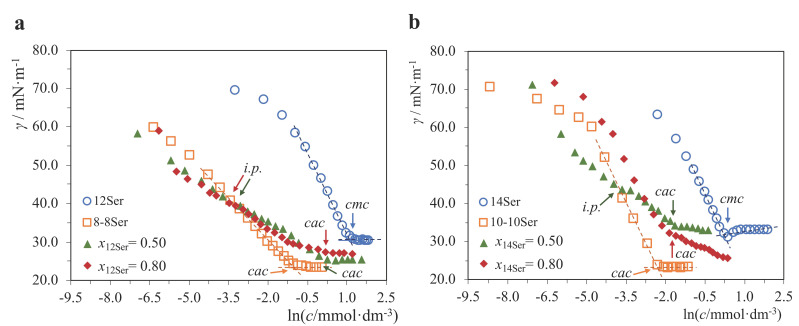
Surface tension plots at 25 °C for the (**a**) 12Ser/8-8Ser and (**b**) 14Ser/10-10Ser catanionic mixtures and their respective neat surfactants.

An important aspect to consider when vesicles are used as membrane models or molecular nanocarriers is their critical aggregation concentration (*cac*). Surface tension measurements were performed for the neat surfactants and for two compositions of the mixtures, *x*_mSer_ = 0.50 (vesicles) and 0.80 (vesicle/micelle coexistence), as shown in Figure 5 and Table 1. For the neat surfactants, the *cac*, the surface tension at that point (*γ_cac_*), as well as the minimum surface area per molecule (*a_s_*), calculated through Equation (1) (Gibbs adsorption isotherm) and Equation (2), were determined. For the mixtures, it is only possible to determine *cac* and *γ_cac_*. The Gibbs isotherm equation applied to a surfactant solution may be expressed as:

(1) $$\Gamma_{s}=-\frac{1}{n_{s}RT}{\left|\frac{d\gamma }{dln\left(c_{s}/c^{o}\right)}\right|}_{T}$$

where *Γ_s_* is the surface excess of surfactant (for zero surface excess of the solvent), *R* is the gas constant, *T* is the temperature, *n_s_* is the number of adsorbed species obtained from the surfactant at the interface (Gibbs pre-factor) and d*γ*/dln(*c_s_*/*c^o^*) is the local slope of the *γ* vs. ln(*c_s_*/*c^o^*) curve (*c^o^* here is the reference concentration, 1 mol·dm^−3^). The minimum surface area per surfactant molecule is calculated from:

(2) $$a_{s}=\frac{1}{\Gamma_{s,max}\;N_{A}}$$

where *N_A_* is Avogadro’s constant and *Γ_s,max_* is the maximum surface excess calculated for the biggest slope of the *γ* vs. ln(*c_i_*/*c^o^*) curve prior to the *cac* inflection point.

For ionic surfactants in neat water, typically the number of adsorbed species *n*_s_ in Equation (1) is equal to 2 (charged amphiphilic ion + respective counterion). However, for 8-8Ser and 10-10ser, due to pH adjustment with KOH, the large excess of small ions should partially screen the interactions at the interface, and hence make n_s_ closer to 1 than to 2 [57,58]. Having this in mind, the *a_s_* values for 8-8Ser and 10-10ser in Table 1 were calculated for both extremes: *n_s_* = 1 and 2. We may reasonably infer that these *a_s_* values are likely closer to 1.2 and 1.0 nm^2^, respectively (Table 1). This is because they are expected to be larger than the areas of 12Ser and 14Ser (both around 0.6 nm^2^), since the double-chained surfactants have bulkier hydrophobic portions, hence requiring more critical area in the adsorbed monolayer. Consistently, the surface tension at *cac*, *γ_cac_*, is significantly lower for 8-8Ser (23.6 mN·m^−1^) and 10-10Ser (23.2 mN·m^−1^), than for 12Ser (30.2 mN·m^−1^) and 14Ser (32.9 mN·m^−1^). This reflects the higher tendency of the double-chained unimers to be surface-adsorbed than the single-chained ones.

As can be seen in Figure 4, the neat surfactants present typical surface tension curves with a breaking point corresponding to the *cac*. 8-8Ser and 10Ser-Ser possess *cac* values of 0.32 and 0.10 mmol.dm^−3^, respectively (Table 1), at adjusted pH = 12, forming vesicles as the aggregation state (we cannot exclude the formation of non-spherical micelles at the *cac*, but as we do not have clarifying evidence, we use the more general designation *cac* instead of *cmc*). 12Ser and 14Ser, which unequivocally aggregate into micelles, have *cmc* values of 3.0 and 1.2 mmol.dm^−3^, respectively (Table 1). The bigger the total chain length of the surfactants, the smaller the *cac or cmc*, as a consequence of the bigger contribution of the hydrophobic effect term to the Gibbs energy of aggregation.

Looking at the surface tension curves of the mixtures, there is a clear *cac*, but also at smaller concentration, a subtle inflection point (indicated as i.p. in the curves), with a very short “plateau”. This type of profile, with two breaking points (i.p. + *cac*) in the surface tension curves, has been reported before for some catanionic mixtures [28,64] and other types of surfactant mixtures [65]. The i.p. is particularly evident in Figure 4b for the 0.50 mixture. The existence of the two points has been interpreted as signaling a critical aggregation event prior to the main one at the *cac*, with the consequence that at the *cac* there is coexistence of two types of aggregates. In both mixtures, for *x*_mSer_ = 0.80, there are indeed coexisting micelles and vesicles; for *x*_mSer_ = 0.50, two vesicle populations coexist (bimodal populations). In both cases, the curve profile would thus be qualitatively justified and consistent with the DLS data. Despite its relevance, we did not investigate this issue any further.

If we now analyze the *cac* of the mixtures, compared to the *cac* or *cmc* of the individual surfactants, several interesting facts arise. For the 12Ser/8-8Ser system, the *cac* values of the 0.50 and 0.80 samples are similar between them, about 40–50% of the *cmc* of 12Ser and about 5–6 times the *cac* of 8-8Ser. This indicates that the presence of the double-chained surfactant does not have a particularly great effect in decreasing the *cac* of this mixture. In other words, the 12Ser/8-8Ser catanionic vesicles form for concentrations of the order of 1.0 mmol·dm^−3^, a value sizably bigger than usual for vesicle-forming systems (≈10^−6^–10^−3^ mmol·dm^−3^). Regarding the *γ_cac_* values of 0.50 and 0.80, they are in between those of 12Ser and 8-8Ser, but for the equimolar composition (≈25 mN·m^−1^) very close to that of 8-8Ser (≈24 mN·m^−1^), suggesting that here the gas–liquid interface is enriched in the double-chained surfactant.

Turning now to the 14Ser/10-10Ser system, the features are fairly different. The 0.50 sample has a *cac* (0.20 mmol·dm^−3^) markedly smaller than that of the 0.80 (0.49 mmol·dm^−3^) and closer to the *cac* of 10-10Ser (0.10 mmol·dm^−3^). This means that, contrary to the previous mixture, the double-chained surfactant here induces a large decrease in *cac*. Moreover, the 14Ser/10-10Ser vesicles form at concentrations much smaller than the 12Ser/8-8Ser vesicles (0.2/1.2 ≈ 6 times), a feature that is interesting for drug delivery applications (the lower the *cac* for vesicles, the more potential cytotoxicity issues are mitigated). The *γ_cac_* values of *x*_14Ser_ = 0.50 and 0.80 are virtually identical to those of 14Ser, and this suggests that the gas–liquid interface is enriched in the single-chained surfactant. Overall, and given that the nature of the headgroups is exactly the same in both mixtures, as well as the asymmetry in chain length (n − m), we can conclude that the total chain length (n + 2m) has a marked effect on the aggregation and interfacial properties of these mixtures.

### 3.4. Discussion

#### 3.4.1. Vesicle Formation and Vesicle/Micelle Transition

We can interpret self-assembly in catanionic mixtures resorting, as a first approach, to the surfactant packing parameter (*P_s_*) model [66]. *P_s_* is defined as the ratio *v_hc_*/(*a_o_ l_c_*), where *v_hc_* and *l_c_* are the volume and critical length of the hydrophobic portion of the molecule and *a_o_* is the critical headgroup area at the aggregate interface. The surfactant’s molecular shape and respective *P*_s_ dictate the type of favored aggregate: for an inverted cone and *P_s_* = 0.33, spherical micelles; for an inverted truncated cone and *P_s_* ≈ 0.33–0.5, cylindrical micelles; for a wedge-shaped amphiphile and *P_s_* ≈ 0.5–1, bilayers disks and vesicles; for a cylinder-like shape and *P_s_* ≈ 1, planar bilayers; and for a truncated cone with *P_s_* > 1, reverse structures. The double-chained surfactants 8-8Ser and 10-10Ser have a *P_s_* ≈ 1 and hence form vesicles/bilayers, as we confirmed (by VELM), while the single-chained 12Ser and 14Ser, with *P_s_* ≈ 0.33 form small micelles, as detected by DLS. 

When the cationic mSer surfactant is gradually added to each mixture (increase in *x*_mSer_), both electrostatic headgroup interactions and chain packing effects take place. Initially, when 0 < *x*_mSer_ ≤ 0.40, the electrostatic association between the two surfactants (caused by coulombic headgroup attractions and, especially, by the large ∆*S* > 0 owing to counterion release [9]) dominates and phase separation in the form of solid formation occurs. This precipitation well below equimolarity is likely aided by the fact that the anionic surfactant is partially protonated (RCOOH) and so less cationic surfactant is necessary for charge neutralization, while the insoluble acid concomitantly aids coprecipitation. When enough net cationic charge is present (as shown by the zeta potential data), i.e., for *x*_mSer_ > 0.40, resolubilization takes place, and the vesicles are still stable, since the effective *P_s_* per surfactant remains is reasonably expected to remain in the range 0.5–1. With further addition of the single-chained surfactant (roughly *x*_mSer_ > 0.70), the excess positive charge and curvature effects (dictated by the intrinsic *P*_s_ ≈ 0.3 of mSer) prevail, and therefore bilayers are no longer the single aggregation state. The system responds in the form of coexisting vesicles and micelles (which implies some non-ideal mixing, as shown by the surface tension data). When *x*_mSer_ > 0.90, only mixed micelles are stable and bilayers disappear from the system, as expected.

In catanionic mixtures, the transition between micelles and vesicles usually occurs via two routes [9]. One route encompasses micelle–vesicle coexistence and limited micellar growth, and is common for surfactants with symmetric chain lengths, such as the DDAB/SDS [25], DTAB/SDS [67], DTAB/12Lys12 [16] and 12Ser/12Lys12 [68] systems. The other path involves the formation of big micelles and is typical of highly asymmetric systems (surfactants with very unequal chain lengths or with chain branching). In this case, upon addition of the micelle-forming surfactant, vesicles gradually solubilize into large rodlike or wormlike micelles and then to small micelles, without any intermediate phase separation. This is the behavior, for example, of the CTAB/SOS mixture (where m − n = 8) [69]. Despite the asymmetry in chain lengths, the behavior of the 12Ser/8-8Ser and 14Ser/10-10Ser systems is more similar to the symmetric than to the asymmetric case, likely because their asymmetry (m − n = 4) is still relatively small.

We can also directly compare these systems to the 16Ser/8-8Ser (m − n = 8, highly asymmetric) and 12Ser/12-12Ser (symmetric) systems previously investigated by us [28], since the surfactant headgroups at stake are exactly the same. For 16Ser/8-8Ser, phase separation takes place in the form of a liquid–liquid equilibrium in a narrow compositional region, and both net anionic and net cationic vesicles appear. The net cationic vesicles, in particular, are monomodal and with <*D*_H_> = 200 nm, and vesicle/micelle coexistence involves large wormlike micelles. The 12Ser/12-12Ser system is dominated by precipitation at room temperature and mixed polydisperse vesicles only form at 60 °C. The 12Ser/8-8Ser and 14Ser/10-10Ser have very different features from both, being intermediate between the highly asymmetric case (sharing with it formation of vesicles and micelle/vesicle coexistence at room temperature) and the symmetric case (sharing solid–liquid phase separation).

#### 3.4.2. Vesicle Stabilization

Several models have been proposed to explain vesicles as equilibrium aggregates in catanionic mixtures (i.e., thermodynamically stable) rather than as a metastable dispersed form of a lamellar liquid crystal [63,69,70]. We will not analyze the issue of vesicle stabilization in detail here, but only make some brief remarks. The elastic surface model for a surfactant film considers that the stabilization of catanionic vesicles derives from two basic alternative mechanisms [69]. In one mechanism, (A), the existence of a non-zero mean spontaneous curvature leads to curvature energy stabilization, yielding small vesicles of low polydispersity, if the normal curvature modulus (*κ*) is high. This is the case of charged and relatively rigid catanionic bilayers. It implies that only narrow mixing ratio ranges (slightly non-equimolar) are optimal for vesicle stability and any deviations dictate an unfavorable bending energy. CTAB/sodium perfluorooctanoate [69], DTAB/12Lys12 [16] and 12Ser/12Lys12 [68] vesicles are presumed to be of this kind. In the other mechanism, (B), implying low values of *κ* (≈ few *k*_B_T), i.e., relatively soft flexible catanionic bilayers, an entropic stabilization takes place leading to big and polydisperse vesicles, and wider surfactant mixing ratios for vesiculation. Here, chain length asymmetry and packing effects of dissimilar hydrocarbon chains contribute to decrease *κ* and promote stabilization. This is assumed to be the case of CTAB/SOS [69], 12Ser/8Lys8 [68] and, significantly, the already-mentioned 16Ser/8-8Ser [28] vesicles. In the 12Ser/8-8Ser system, we found monomodal vesicles for a narrow mixing ratio region, with a constant size and an average composition of about *R*_+/−_ = 2:1 (DLS data). These features are more akin to those of a curvature stabilization mechanism, (A), than to an entropic one, (B). In contrast, the more polydisperse 14Ser/10-10Ser vesicles and the variation in vesicle size depending on sample composition seem to underpin an entropic stabilization mechanism.

## 4. Conclusions

Herein, we showed that two mixed cationic/anionic surfactant systems based only on serine-derived surfactants, 12Ser/8-8Ser and 14Ser/10-10Ser, are able to form stable vesicles at room temperature, with no previous pH adjustment, under some specific mixture compositions (the vesicles are always net positively charged). One first general conclusion is that these serine-based surfactants provide a source for preparing model, robust biomimetic vesicles than can be further explored for bio-related applications, namely for drug and (given their positive charge) gene delivery. These two mixtures share an identical surfactant chain length asymmetry (four carbon atoms), but different total alkyl chain size (28 versus 34 carbon atoms). A second general conclusion is that even if we retain chain length asymmetry, a relatively small change in total chain length has meaningful effects on the vesicle features. The 12Ser/8-8Ser vesicles exist either as bimodal or unimodal populations, in a wider compositional range. However, the unimodal vesicles seem to have a specific average composition of *R*_+/−_ ≈ 2:1. They also have <*D*_H_> ≈ 250 nm, *ζ* = +35–40 mV and pH ≈ 7–8, forming at a total surfactant concentration ≥ 1 mmol·dm^−3^. Overall, they show interesting characteristics for biomolecular delivery. The 14Ser/10-10Ser vesicles instead form for a narrower compositional region and appear only as bimodal, polydisperse populations (80 and 300 nm), *ζ* = +45–50 mV and pH ≈ 9–10, forming at smaller concentrations (≥0.2 mmol·dm^−3^).

## Figures and Tables

**Table 1:** Interfacial parameters obtained for the neat surfactants and catanionic mixtures with specific compositions, at 25 °C, from surface tension data.

System	*x*_mSer_	pH	*cac*/mmol·dm^−3^	*γ_cac_*/mN·m^−1^	*a_s_*/nm^2^
8-8Ser	0	12.0 ^(1)^	0.25 ± 0.04	23.6	1.2 ± 0.1 (*n_s_* = 1) 0.6 ± 0.1 (*n_s_* = 2)
12Ser	1	5.3	3.0 ± 0.1 ^(2)^	30.2	0.56 ± 0.05
12Ser/8-8Ser	0.50	9.8	1.2 ± 0.1	25.2	-
12Ser/8-8Ser	0.80	7.0	1.4 ± 0.1	27.1	-
10-10Ser	0	12.0 ^(1)^	0.10 ± 0.02	23.2	1.2 ± 0.1 (*n_s_* = 1) 0.6 ± 0.1 (*n_s_* = 2)
14Ser	1	4.6	1.2 ± 0.1 ^(2)^	32.9	0.55 ± 0.06
14Ser/10-10Ser	0.50	9.2	0.20 ± 0.04	32.2	-
14Ser/10-10Ser	0.80	5.8	0.49 ± 0.02	32.5	-

^(1)^ Adjusted with KOH. For all other systems, the pH is the natural one. ^(2)^ Values are specifically critical micelle concentration, *cmc*. For 14Ser, measured at 30 °C.
